# Supplementary material for: Blood Flow Measurements Enable Optimization of Light Delivery for Personalized Photodynamic Therapy
Source: Cancers (Basel). 2020 Jun 15;12(6):1584. doi: 10.3390/cancers12061584 (PMC7353010; doi:10.3390/cancers12061584)
Supplement: Supplementary file 1 [file cancers-12-01584-s001.pdf]

Supplementary Materials

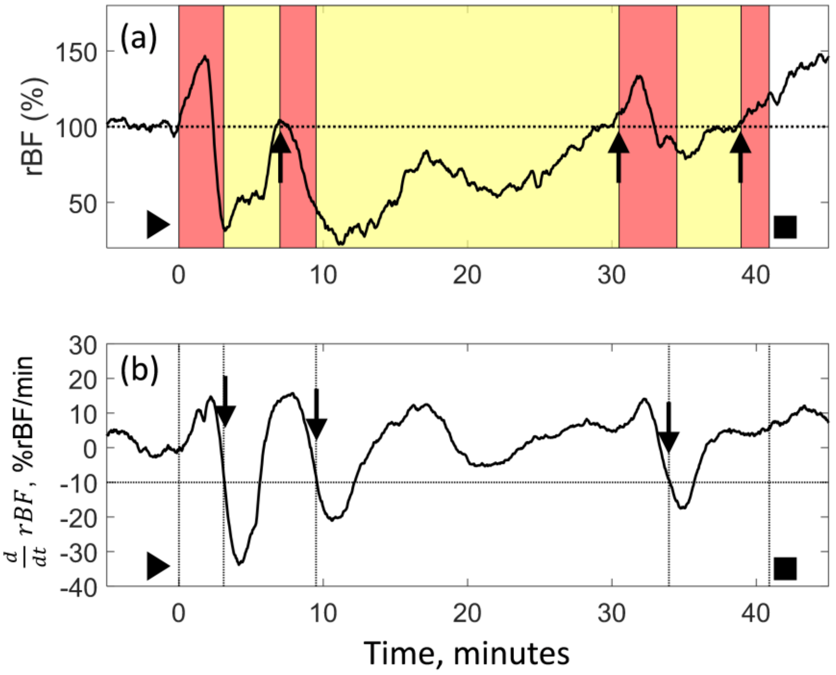

Figure S1. (**a**) Representative relative blood flow traces of RIF tumor for BFI-Irrad, and (**b**) the corresponding time derivative/slope of rBF computed in real-time from data in (a). Light delivery is indicated in (a) and is shaded in red or yellow for illumination at 150mWcm^{-2} or 25mWcm^{-2}, respectively. ▶ and ■ indicate the initiation and completion of light delivery, respectively. Downward pointing arrows in (b) indicate instances when rBF decreases more than 10% per minute (or rBF slope ≤ -10%rBFmin^{-1}) and irradiance is attenuated from 150mWcm^{-2} to 25mWcm^{-2}. Upward pointing arrows in (a) indicate instances when rBF(t) recovers to above $rBF_{baseline}$, and irradiance is increased from 25mWcm^{-2} to 150mWcm^{-2}. .

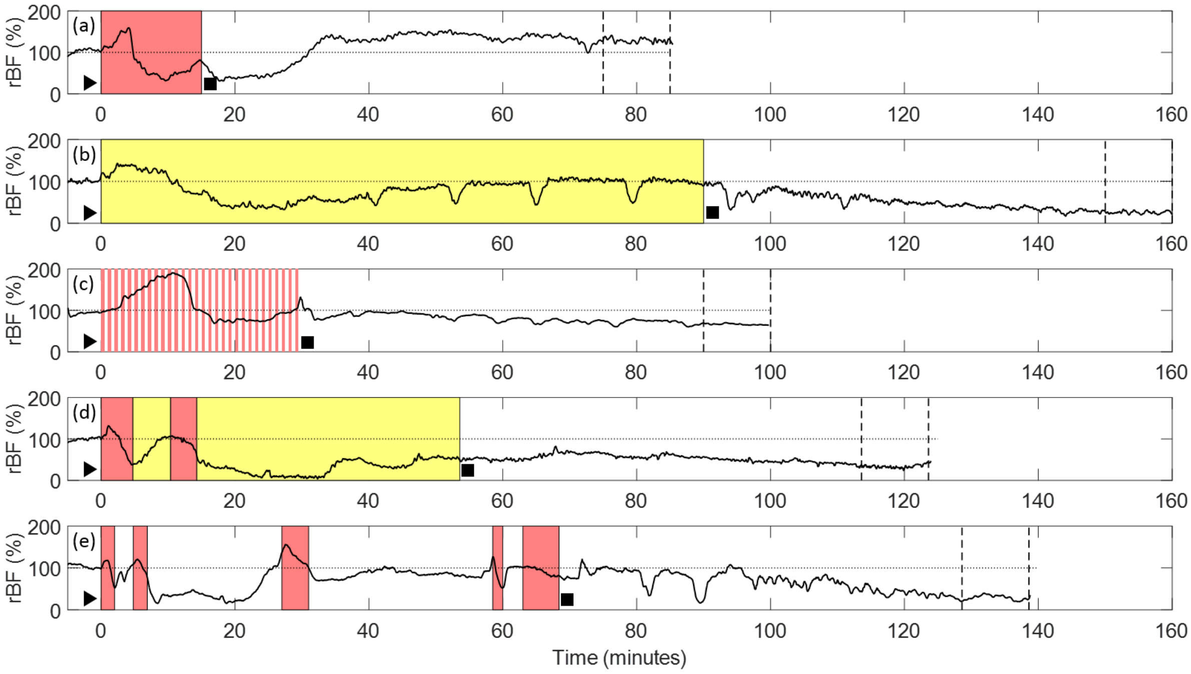

Figure S2. Representative blood flow traces of RIF tumors for the pre-PDT through post-PDT timeframe: (**a**) 150mWcm^{-2} -continuous; (**b**) 25mWcm^{-2}-continuous; (**c**) standard 150 mWcm^{-2}-fractionated illumination; (**d**) BFI-Irrad; (**e**) BFI-Frac. ▶ and ■ indicate the initiation and conclusion of light delivery, respectively. Light delivery is shaded in red or yellow for illumination at 150mWcm^{-2} or 25mWcm^{-2}, respectively, with unshaded areas representing periods without illumination. Vertical dashed lines indicate 60 and 70 minutes after completion of light delivery for each treatment condition.

Table S1. Summary of median (95% confidence interval) percent of complete response and time-to-400 mm^3 for treatments of RIF tumors using each of the standard and BFI-PDT illumination schemes.

Type of PDT	Group	Percent of complete response (%) Median (95% CI)	Time-to-400 mm^3 (days) Median (95% CI)
Standard	150 mWcm^{-2}- continuous	0	11 (6, *)
	25 mWcm^{-2}- continuous	30 (7, 65)	19.6 (12, *)
	150 mWcm^{-2}- fractionated	22 (2, 48)	18 (12, *)
Blood-flow informed	BFI-Irrad	40 (12, 74.5)	29 (18, *)
	BFI-Frac	56 (18, 91)	39 (18, *)

*Unable to estimate due to insufficient events. Response rates align with Kaplan Meier curves of Figure 5 (which incorporate censoring for fractionated treatments that produced morbidity).

Table S2. Summary of median (95% confidence interval) percent of complete response and time-to-400 mm^3 for treatments of AB12 tumors using 150 mWcm^{-2}-continuous, 25 mWcm^{-2}-continuous and BFI-Irrad illumination schemes.

Group	Percent of complete response (%) Median (95% CI)	Time-to-400 mm^3 (days) Median (95% CI)
150 mWcm^{-2}- continuous	33 (7, 71)	20 (6, *)
25 mWcm^{-2}- continuous	89 (52, 100)	>90 (*, *)
BFI-Irrad	100 (69, 100)	>90 (*, *)

*Unable to estimate due to insufficient events.
